# Worldwide dietary patterns and their association with socioeconomic data: an ecological exploratory study

**DOI:** 10.1186/s12992-022-00820-w

**Published:** 2022-03-12

**Authors:** Gabriel Gonçalves da Costa, Giovanna da Conceição Nepomuceno, Alessandra da Silva Pereira, Bruno Francisco Teixeira Simões

**Affiliations:** 1 School of Nutrition, Federal University of The State of Rio de Janeiro (UNIRIO), Urca 296, Rio de Janeiro, Rio de Janeiro Brazil; 2 Quantitative Methods Department, Avenue Pasteur, Federal University of the State of Rio de Janeiro (UNIRIO), Urca 458, Rio de Janeiro, Rio de Janeiro Brazil

**Keywords:** Multivariate analysis, Western diet, Western dietary pattern, *Food supply*, Food balance sheets

## Abstract

**Background:**

Dietary patterns are associated with health outcomes and environment sustainability, having socioeconomic drivers. This ecological study aims to identify dietary patterns in food availability data at the global level using multivariate statistical methodology, to associate the identified dietary patterns with socioeconomic data and to analyze the adequacy of the applied multivariate statistical methods for this purpose.

**Methods:**

Principal Component Analysis was applied to median values of times series of food availability data of 172 UN registered countries available at FAOSTAT database in Food Balance Sheets section in a sectional manner, after calculating median values of time series for each food group and country. Principal Components were associated with socioeconomic data available from the World Bank database. Sensitivity analyses were realized to verify the stability of dietary patterns through five different times.

**Results:**

Five principal components were identified in the median values of each time series, each characterizing a possible dietary pattern. The first one, a westernized dietary pattern, was composed of energy-dense and processed foods, foods of animal origin, alcoholic beverages, but also, albeit less, by vegetables, fruits and nuts, being associated with income, urbanization and trade liberalization. This westernized pattern was characterized more animal origin and processed foods, such as vegetable oils, alcoholic beverages and stimulants yet preserving unprocessed and regional foods. The other dietary patterns were three agricultural patterns characterized more by regional foods, especially starchy staples, and one coastal dietary pattern composed of fish and seafoods, being associated with GINI index, poverty, and female labor force. Sensitivity analyses demonstrated the stability of dietary patterns.

**Conclusions:**

Principal Component Analysis was adequate to identify dietary patterns in food availability data. A westernized dietary pattern was identified, being associated with income, urbanization, and trade liberalization. This association did not occur for the remain of the dietary patterns identified, these being less driven by economic development.

## Background

According to the Food and Agriculture Organization of the United Nations (FAO), Food Balance Sheets (FBS) [1], presents the quantity of food items available for human consumption, containing data of food availability (or food disappearance). It is calculated from food supply (food production plus imports minus raw quantity produced in the country that is exported) after factors such as food waste in the supply chain and industrial usage are subtracted from the country’s domestic supply of a given food commodity. Per capita food availability for human consumption, calculated at the country level, presents a comprehensive picture of the patterns of food supply of a given country during a specified reference period and is associated with household food availability and individual food consumption, this latter assessed by food frequency questionnaires and the 24-h dietary recall in studies with representative samples of the country’s population [2, 3, 72].

In this sense, food availability has increased in many countries in recent decades, not only in total raw quantity but also in variety. There has been a transition from a low variety of starchy staples to a greater variety of foods, with an increase in the availability of animal origin and energy-rich foods, as well as the level of food processing. This has resulted in changes in dietary patterns of populations around the world and is part of the nutrition transition, as well as the process of westernization and globalization of diets [4–7].

Currently, a western dietary pattern characterizes the diet of most of the global population. This dietary pattern is associated with health and environmental problems, a relationship that will become stronger if historical trends of food availability remain in the future. In the health dimension, there is for example its association with non-communicable chronic diseases. For environmental sustainability, there is the discussion about the environmental impact of food of animal origin and of processed food [8–12].

Even if westernization and globalization lead to homogenization of diets towards a western pattern, the scenario outlined above occur heterogeneously in different regions. Food availability and consumption depend on a myriad of factors, such as economic, political, cultural, social, or even biophysical, which eventually interact with each other [13, 14]. For example, socioeconomic drivers of the availability of western and processed food are income, urbanization, trade liberalization and economic development in general [4, 6, 12, 15]. In this context, there is empirical evidence that some regions preserved traditional foods more than others in response to the westernization process and to nutrition transition [4, 6, 16]. In this sense, a western diet may not be uniform globally, but generally is characterized by an increase in the availability of animal origin and energy-rich foods, as well as the level of food processing [4, 5, 7].

Socioeconomic variables and food availability have been already previously studied at the national or continental level, with multiple countries and/or continents being analyzed simultaneously with substantial historical coverage [5, 17]. Even if food availability as calculated from FAO Food Balance Sheets was already used to identify both a priori and a posteriori dietary patterns in the literature, analyses with multivariate statistical tools to identify a posteriori dietary patterns in these data at the global level may still be lacking [10, 18]. Additionally, it is known that diets across the globe are becoming increasingly homogeneous, a direct feature of the westernization process, and there is evidence that the nutrition transition is growing at a faster pace in many regions of the globe [19].

In food availability data (including food supply from FBS and household food availability), dietary patterns can also be called dietary availability patterns, even if food supply is not able to measure food consumption and diet directly [2, 3, 10, 20, 21]. The nomenclature “dietary pattern” is mostly referred to statistical patterns derived a posteriori with multivariate statistical methodologies from food data, i.e., food supply (or food availability), household food availability or food consumption [20, 21].

The objective of this study was to identify dietary patterns in food availability data at the global level, to associate these patterns with socioeconomic data and to analyze the adequacy of the multivariate statistical methods applied.

## Methods

### Study design, data collection and data characteristics

Food availability data in kg/capita/year (item: food supply quantity kg/capita/yr) between 1961 to 2013 (representing time series with 53 data observations units) from Food Balance Sheets of each of the 172 countries registered at United Nations available at FAOSTAT database [22] were downloaded for this ecological study. More recent data from food balance sheets from 2014 to 2017 are already currently available but still in a preliminary form, with distinct methodology than of the previous years [23].

Food availability methodology has been described elsewhere [1]. It is calculated from food supply (food production plus imports minus raw quantity produced in the country that is exported) after factors such as food waste in the supply chain and industrial usage of the commodity are subtracted from the domestic supply. A distinction is made between the quantities exported, fed to livestock, used for seed, put to manufacture for food use and non-food uses, losses during storage and transportation, and food supplies available for human consumption. The per caput supply of each such food item available for human consumption (food availability per capita) is then obtained by dividing the respective quantity by the related data on the population partaking on a given country [2, 3].

Food availability data were downloaded by 18 food groups: Meat, Milk – Excluding Butter, Eggs, Animal Fats, Offals, Alcoholic Beverages, Sugars & Sweeteners, Vegetable Oils, Pulses, Starchy Roots, Cereals – Excluding Beer, Stimulants, Fish – Seafood, Vegetables, Oilcrops, Treenuts, Fruits – Excluding Wine and Spices, with one dataset for each food group. Sugar Crops, Aquatic Products, and Miscellaneous were not analyzed due to the high frequency of missing time series and missing values.

### Data analysis

Given the non-gaussian distribution of many of the time series available and for obtaining one single value of food availability by food group for each country, median values for each time series were obtained, since it represents a robust estimate of asymmetric frequency distributions. Thereafter, all values in kg/capita/day were standardized to z-scores.

Multivariate normality was analyzed by the Shapiro-Wilk Multivariate Normality Test. Principal Component Analysis (PCA) was used to identify dietary patterns, using Spearman correlation for the derivation of principal components and Kaiser’s Rule as criteria for the number of principal components to be retained (then retaining all principal components with eigen values higher than one unit). For the adequacy of PCA, Kaiser-Meyer-Olkin (KMO) and Bartlett’s Tests were performed.

Each principal component retained was then associated with the median values of each time series of each food group for each country in raw data (kg/capita/year) to characterize each dietary pattern. Food groups with significant (*p* <  0.05) and positive correlation with one principal component were considered to contribute to its construct, characterizing one dietary pattern.

In PCA, high-dimensional and linear dependent data are reduced to fewer variables, the Principal Components. In this transformation, there is a minimum loss of variance. The first principal component explains the greater share of variance and the reminiscent share is explained in a decrescent manner by each subsequent principal component. The number of principal components to be retained is usually analyzed by the Kaiser’s Rule when the correlation matrix is considered [20, 21, 24, 25].

Each principal component was then correlated using the Spearman method with socioeconomic data downloaded at the World Bank database, given the non-parametric distribution of each of the principal components (Shapiro-Wilk Normality Test, *p* <  0.05). Subsequently, quantile regressions with the median value as conditional quantile (tau = 0.5) between the first principal component and socioeconomic data were performed.

Socioeconomic data for GNI per capita in constant 2010 dollars [26], Poverty Headcount Ratio [27], Urban Population [28], Trade – % of GDP [29], Labor force – female [30] and GINI index [31] were downloaded. Each socioeconomic variable was also available in years, and medians of each variable (also from time series of 1961 to 2013 – the same temporal gap that of food availability data) were also obtained for each country.

A cutoff point of 1,90 dollars per capita per day with 2011 Purchasing Power Parity (PPP) values was utilized for poverty. Trade – % of GDP was transformed into raw data using GDP per capita (2010 dollars) as reference (32).

Countries were also classified by income level, using the World Bank Country classification by income level 2019-2020. Considering this classification, 23 countries were classified as low-income, 45 as lower middle-income, 49 as upper middle-income and 54 as high-income.

To classify the magnitude of correlation, it was considered 0.0 to 0.3 (or 0.0 to − 0.3) for weak correlation, 0.3 to 0.7 (or − 0.3 to − 0.7) for moderate correlation and 0.7 to 1.0 (or − 0.7 to − 1.0) for strong correlation. All data and analyses code can be assessed at Open Science Framework [33] and can also be provided by contact by e-mail with the first author.

Given that the main analysis was a sectional one being composed of median values of each time series for each food group and country, sensitivity analyses were performed to verify the stability of dietary patterns considering five times spaced by 10 to 9 years. This spacing was considered, hoping that the pattern does not change significantly in shorter periods. In this analysis, dietary patterns were characterized by food groups that had a more significant correlation with the main component.

### Statistical softwares

Software R [34] version 3.4.4 was used for data analysis, along with the packages: stats [34], ggplot2 [35], and factoextra [36]. For the multivariate normality test, the package mvnormtest [37] was used. For PCA analysis, the prcomp function was used, being the correlation matrix of the original data considered when performing the PCA.

## Results

### Adequacy of principal component analysis

Given the non-gaussian distribution of the data (Shapiro-Wilk Multivariate Normality Test, *p* <  0.05), and KMO (Overall MSA = 0.77) and Bartlett’s Test (*p* <  0.01), PCA was the adequate technique to be applied.

The standard deviation (square root of the eigen value of each principal component) and the proportion of variance of each principal component can be seen in Table 1, being the proportion of variance of each component the eigen value divided by total variance (sum of eigen values). In the present analysis, 69.55% of the variance-covariance structure of the data (that was standardized in z-scores) could be explained by the retained principal components and five principal components with eigen values more than one unit were retained by Kaiser’s rule [24].

**Table 1:** Standard deviation, individual proportion and cumulative proportion of explained variance of each retained principal component

Principal Components Statistics	PC1	PC2	PC3	PC4	PC5
**Standard Deviation**	2.5271	1.5013	1.21352	1.13411	1.05841
**Proportion of Variance**	0.3548	0.1252	0.08181	0.07146	0.06223
**Cumulative Proportion**	0.3548	0.48	0.56184	0.63329	0.69553

The correlation between the principal components obtained and the food availability raw data can be seen in Table 2, where food groups with positive and significant correlations characterize the dietary pattern, expressed as a principal component.

**Table 2:** Statistically significant correlations between food availability data and principal components

Food groups	PC1	PC2	PC3	PC4	PC5
Alcoholic Beverages	0.68	ns	ns	ns	ns
Animal Fats	0.84	ns	ns	ns	ns
Cereals - Excluding Beer	ns	0.91	ns	ns	ns
Eggs	0.87	ns	ns	ns	ns
Fish - Seafood	0.34	−0.49	−0.46	0.23	ns
Fruits - Excluding Wine	0.56	−0.44	ns	−0.42	−0.26
Meats	0.88	ns	ns	ns	ns
Milk - Excluding Butter	0.88	ns	ns	ns	ns
Offals	0.65	ns	0.3	ns	0.22
Oilcrops	ns	−0.38	−0.35	ns	ns
Pulses	−0.4	ns	0.27	−0.5	−0.3
Spices	ns	ns	−0.35	ns	−0.49
Starchy Roots	ns	−0.57	ns	−0.12	0.5
Stimulants	0.87	ns	ns	ns	ns
Sugars & Sweeteners	0.8	ns	ns	ns	−0.36
Treenuts	0.53	0.25	−0.4	−0.29	0.22
Vegetable Oils	0.65	ns	−0.21	−0.27	ns
Vegetables	0.62	0.38	−0.38	−0.23	ns

ns equals to non-significant associations

### Dietary patterns and their associations with socioeconomic variables

In Table 3, correlations of the dietary patterns with socioeconomic data can be visualized.

**Table 3:** Correlations between socioeconomic data and principal components

Socioeconomic Variables	PC1	PC2	PC3	PC4	PC5
GINI Index	−0.45	−0.22	0.29	ns	ns
GNI per capita in constant 2010 dollars	0.89	ns	ns	ns	ns
Labor force – female	ns	−0.27	ns	ns	0.43
Poverty Headcount Ratio	−0.86	ns	0.2	ns	ns
Urban Population	0.88	ns	ns	ns	ns
Trade (% of GDP)	0.68	ns	ns	ns	ns

ns equals to non-significant associations

The first principal component (PC1) identified a westernized dietary pattern, with a positive correlation with GDP per capita, % of urban population, and Trade - % of GDP, all socioeconomic variables that are proxy of socioeconomic development [38]. It was characterized by energy-dense and processed food groups (i.e., sugars & sweeteners, vegetable oils and alcoholic beverages), foods of animal origin, but also, albeit less, by vegetables, fruits, and nuts. This principal component explained 35.48% of the variance of the data, with a standard deviation of 2.53.

In the analysis stratified by income country classifications, the correlation between PC1 and income (GNI per capita) was 0.34 (95%CI: − 0.08-0.66, *p*-value = 0.14), 0.62 (95%CI: 0.40-0.78, p-value < 0.01), 0.41 (95%CI: 0.14-0.62, p-value < 0.01) and 0.65 (95%CI: 0.46-0.78, p-value < 0.01) in low-income, lower middle-income, upper middle-income, and high-income country classifications, respectively. The correlation between PC1 and urbanization (% of urban population) was 0.40 (95%CI: − 0.008-0.70, *p*-value = 0.062), 0.68 (95%CI: 0.48-0.81, p-value < 0.05), 0.59 (95%CI: 0.37-0.75, p-value < 0.01) and 0.43 (95%CI: 0.19-0.63, p-value < 0.01) in low-income, lower middle-income, upper middle-income, and high-income country classifications, respectively. The correlation between PC1 and trade liberalization (Trade - % of GDP) was − 0.1 (95%CI: − 0.49-0.33, *p*-value = 0.67), 0.20 (95%CI: − 0.1-0.47, p-value = 0.20), 0.23 (95%CI: − 0.06-0.48, p-value = 0.13) and 0.58 (95%CI: 0.36-0.73, p-value < 0.01) in low-income, lower middle-income, upper middle-income, and high-income country classifications, respectively.

In Table 4, the results of the quantile regressions demonstrate that income was statistically significant associated with PC1 even after adjustment for urbanization and trade liberalization.

**Table 4:** Quantile regressions between PC1 and socioeconomic variables

Factors	Beta	Standard error	t value	***p***-value
**Model 1**				
(Intercept)	−3.91440	0.34327	−11.40326	< 0.001
GNI per capita in constant 2010 dollars (US$)	0.00007	0.00002	2.98405	0.00337
Urban Population (% of total population)	0.06356	0.00959	6.63113	< 0.001
Trade (% of GDP in US$)	0.00000	0.00000	1.18707	0.23727
**Model 2**				
(Intercept)	−3.82618	0.35792	−10.69018	< 0.001
GNI per capita in constant 2010 dollars (US$)	0.00009	0.00003	3.56881	< 0.001
Urban Population (% of total population)	0.05938	0.01042	5.69800	< 0.001

The second principal component (PC2) an agricultural dietary pattern, with a strong and positive correlation with the cereal’s food group, a moderate and positive correlation with the group of vegetables, a positive and weak correlation with the group of Treenuts, and with no significant and positive correlation with any of the socioeconomic variables under study. Given its moderate and positive correlation with the group of vegetables, this dietary pattern was classified as a transitional agricultural dietary pattern. This principal component explained 12.52% of the variance of the data, having a standard deviation of 1.50.

The third principal component (PC3) also identified an agricultural dietary pattern, with positive and weak or moderate correlations with the food groups of Offals and Pulses, respectively. This principal component had positive and significant correlation with Poverty Headcount Ratio and GINI index. This principal component explained 0.08% of the variance of the data, with a standard deviation of 1.21.

The fourth principal component (PC4) identified a coastal dietary pattern, with a positive and weak correlation with the food group of Fish – Seafood and with no significant and positive correlations with any socioeconomic variable. This principal component explained 0.07% of the variance of the data, with a standard deviation of 1.31.

The fifth and last principal component (PC5) identified another agricultural dietary pattern, with a positive and moderate correlation with the food group of Starchy Roots. This principal component had a positive and significant correlation with the female labor force variable. This principal component explained 0.06% of the variance of the data, with a standard deviation of 1.05, being the last principal component with standard deviation and eigen value higher than one unit, and hence the latter retained after applying the PCA.

In Table 5, dietary patterns are characterized by decade. This sensitivity analysis demonstrates that dietary patterns identified above in a sectional manner with the use of median values were stable through time.

**Table 5:** Characterization of dietary patterns by year

Year	PC1	PC2	PC3	PC4	PC5
1961	Milk, Eggs, Animal Fats, Stimulants, Meat	Cereals, Vegetables, Treenuts	Fish, Treenuts, Starchy Roots, Oilcrops	Pulses, Fish, Sugar, Fruits	Sugar, Fruits, Starchy Roots, Pulses
1970	Milk, Eggs, Stimulants, Meat, Animal Fats	Cereals, Starchy Roots, Oilcrops, Fish and seafood	Fish, Treenuts, Vegetables, Vegetables Oils	Pulses, Fruits, Fish, Oilcrops	Fruits, Pulses, Sugar, Starchy Roots
1980	Milk, Eggs, Meat, Animal Fats, Stimulants, Sugar	Cereals, Starchy Roots, Oilcrops, Fruits	Fish, Treenuts, Oilcrops, Vegetables, Vegetable Oils	Pulses, Fish, Fruits, Oilcrops	Fruits, Pulses
1990	Milk, Eggs, Meat, Stimulants, Animal Fats, Alcoholic Beverages	Cereals, Starchy Roots, Vegetables, Oilcrops	Fish, Oilcrops, Treenuts, Vegetables, Fruits	Pulses, Fruits, Fish, Oilcrops	Sugar, Starchy Roots, Fruits
2000	Milk, Eggs, Meat, Stimulants, Animal fats, Sugar	Cereals, Starchy Roots, Vegetables, Oilcrops	Fish, Treenuts, Fruits, Oilcrops	Pulses, Fish, Fruits, Starchy Roots	Starchy Roots, Sugar, Treenuts and Vegetables

In PC1, the westernized dietary pattern, sugar and alcoholic beverages begin to appear in the late 80’s and 90’s. In PC2, cereals were always present, along with vegetables. This principal component was characterized by a transitional agricultural one in the analysis of the median values because it contained already a more variety of foods than the other agricultural patterns (PC3 and PC5). PC4, the coastal dietary pattern, always presented fish in its composition, with varying levels of traditional foods. PC5, the last principal component retained, presented pulses until the decade of the 80’s, after that it became to transition to a more varied pattern. PC3 was the least consistent and stable principal component between the sectional analysis and this temporal analysis.

## Discussion

In the present ecological study Principal Component Analysis was the chosen statistical multivariate method to identify dietary patterns given that the data were not normally distributed, even after z-scores standardization of the median values of each time series, and given its adequacy analyzed by KMO and Bartlett’s Tests. Factor analysis, other multivariate statistical technique with similarities with PCA, can only be applied when the data follow a multivariate normal distribution [24, 25]. The multivariate normality was assessed by the Shapiro-Wilk multivariate normality test, with a *p* < 0.05 indicating that the data were not normally distributed (the null hypothesis of this statistical test is that the data are normally distributed). We decided to label this study as an ecological study based on other previous similar studies in the literature [10, 39] and this is in agreement with the good practices of reporting of observational studies [40]. Other studies with different objectives also were labelled as ecological using food balance sheets data [41]. The present study does sectional country analyses using median values of time series of food availability data in countries with sensitivity analyses through 5 different time points. In ecological studies the unit of observation is the population or community, and time trend analyses are also valid [42].

In a literature review that analyzed studies between 1980 and 2012, no study was found to use Principal Component Analysis nor any other common multivariate statistical analysis (i.e., Factorial Analysis, Reduced Rank Regression and Cluster Analysis) in Food Balance Sheets data [8]. Of note, there was two studies that performed cluster analysis in Food Balance Sheets data, one published in 2013 [10] and the other in 2015 [39].

We used the Kaiser’s method as an objective criteria to retain the number of principal components [24]. In a literature review of multivariate statistical methods to identify a posteriori dietary patterns, in approximately 30% of the time between 4 and 5 principal components were usually retained when using PCA [8]. It is common in the literature that subjectivity remains involved in this process of labelling dietary patterns, such as retaining patterns due to their interpretability (i.e., if the pattern can be clearly labelled) and not using objective statistical criteria such as the Kaiser’s rule [43].

The westernized dietary pattern is associated with socioeconomic variables that are proxy of economic development [4, 38]. Previously, with distinct methodology, similar results were found in dietary patterns derived by multivariate statistical techniques using food availability data [10]. This first pattern identified in the analysis (PC1) is the one that most explains the variability of the data.

The second principal component, identifying a transitional agricultural dietary pattern, is characterized as transitional accordingly to previous studies [10, 39], capturing a pattern characterized by the expansion stage of the nutrition transition, where vegetables also appear alongside with starchy staples in the dietary pattern, occurring before the substitution stage where a dietary pattern switches to a mainly western one [4, 39, 44].

Income influence food availability as stated by Bennett’s Law. With rising incomes, there is a shift in diets, from a starchy staple mainly scenario to a more varied one, with the presence of more calorie-rich and western foods [13, 15, 45]. Also, Engel’s Law establishes that not only income needs to be accounted for when assessing drivers of dietary patterns, but also income distribution [46]. For example, growing demand for meat increases in a U-shaped curve, and after some amount of income, the demand for meat stagnates or even declines, this association being also dependent on geographic and cultural factors [4, 44]. That is, there is a saturation point where demand for food availability no longer responds to growing incomes [4, 44, 46].

Urbanization has a similar role in driving dietary patterns. Food availability changes occur first in urban regions, only occurring in rural regions if there is income increase [6]. Although more debate is needed in the literature, there is some evidence that income and urbanization have different roles in diet, the first increasing variety of foods in the menu, the latter changing regional traditions of diets and causing westernization [16]. There is also evidence that the nutrition transition is occurring at lower levels of income and urbanization than previously thought [17].

It is important to note that further exploratory analyses were performed to assess the correlation between the principal components (dietary patterns) and the socioeconomic variables by country income classification (World Bank Country Classification by income level 2019-2020) to assess the confounding effect of income in the association between the dietary patterns and other socioeconomic variables such as trade liberalization and urbanization. These analyses could only control but not remove entirely the confounding effect of income, because country income classifications are not continuous variables but categorical ones, and each income classification compromises a range of income values. The correlation between PC1 (the westernized dietary pattern) and income was not entirely removed in low-income, lower middle-income, upper middle-income, and high-income country classifications, respectively, suggesting that the influence of income on the dietary patterns was not entirely removed after stratification.

One can argue that urbanization and income can fundamentally indicate one same phenomenon, that is, of economic development. Their correlation was positive and strong (r = 0.8, 95%CI: 0.74-0.85, *p* < 0.05), however, urbanization had positive and moderate/strong correlations with the westernized dietary pattern in different subgroups of country income classifications, suggesting an independent association of this variable. Both urbanization and income maintained statistically significant associations with the westernized dietary patterns after adjusting for trade liberalization in the quantile regression. Urbanization maintained its association with the westernized dietary pattern after adjusting only for income in Table 4 (model 2).

Trade liberalization, represented (as a proxy) in this study by the variable Trade – % of GDP [29], can change the commodities market, where countries with greater insertion in the global trade market present more dependence on foods of the western dietary pattern. Marketing and the market of foods exerts an influence on consumption of the foods that characterize this pattern [47]. Trade liberalization was also strong and positively correlated with income (r = 0.63, 95%CI: 0.53-0.71, *p* < 0.05), losing statistical significance with the westernized dietary pattern after adjusting for urbanization and income. In the analysis of income strata, trade liberalization was only statistically significant correlated with this dietary pattern in high-income countries.

Even if correlation is not equal to causation, trade liberalization had a stronger correlation with the westernized dietary pattern in high-income countries, suggesting that the play of income comes first as of trade liberalization in the westernization process. The correlation between the westernized dietary pattern and income is higher in the transition between low-income to lower middle-income. Also, urbanization has stronger correlations comparing middle-income to low-income countries, suggesting that the play of the urbanization process in westernization of dietary patterns comes in lower levels of income when compared to trade liberalization. It is expected that countries with higher income have higher availability and variety of food groups, and is in accordance with Bennet’s Law [45]. It may be difficulty to identify if whether the observed patterns are demand or supply driven. It is possible that a mix between these drivers occur, since marketing of foods also affects food availability and consumption [47]. Foreign direct investment is also a driver of greater food availability, food processing as well as the nutrition transition [48]. One previous study found an ecological correlation between the density of fast food restaurants in a country with the prevalence of obesity [49].

These socioeconomic variables did not presented positive and significant correlations with the agricultural dietary patterns identified in this study in a similar manner as in previous studies [10, 39], since these patterns are mainly composed by starchy staple foods, and these foods are not correlated with economic development [4, 7, 17].

Fish and seafood are present both in PC1 and PC4. This food group can be driven both by economic development and by geographical factors. In PC1, fish and seafood are driven by economic development, being this food group present in a westernized dietary pattern, a pattern with higher amount of protein and foods of animal origin [14, 17]. The rise in income is a main driver for the growth of food availability of fish worldwide. In PC4, it may be driven by geographical factors since this pattern is not associated with income and urbanization and PC4 does not have significant correlation with any other socioeconomic variable under study. Fish and seafood compromise an important food group for food security and human nutrition. In low-income populations that depend heavily on a narrow range of calorie-dense staple foods and can consume fish locally available, fish can represent a much-needed means of nutritional diversification that is relatively cheap. Within countries, consumption is higher in coastal marine and inland water regions [7, 14, 17]. When plotting PC4 in a world map, it is possible to see that this principal component is more present in countries with litoranean regions (data not shown). In a FAO report, fish contribute mostly to the protein supply in countries with litoranean regions as well [50]. Traditional dietary patterns are common in multivariate analysis in studies of nutritional epidemiology [8]. A traditional pattern preservers regional characteristics of their respective regions [8]. Previously, the nomenclature traditional-coast has already been used to describe a dietary pattern characterized by fruits, nuts, starchy plants, and fish [51]. Given that this dietary pattern is not associated with any socioeconomic variable studied and it is only associated with fish and seafood, it may be driven by geographical and cultural factors.

### Agricultural patterns – regional and cultural differences

Given these results, none of the agricultural dietary patterns identified were characterized by more than one starchy staple (cereals, pulses, or starchy roots food groups). In this sense, other factors than the socioeconomic ones could explain differences between PC2, PC3, and PC5 (all these three being characterized as agricultural dietary patterns), as for example, cultural and regional factors. The adherence to dietary patterns are dependent also of regional and demographical characteristics [52, 53]. In Africa, there is a great variety of food spending patterns, especially starch staples, and this can explain why the food groups of cereals, starchy roots, and pulses did not share a principal component. Regions where agricultural dietary patterns prevail are of low-income and high food insecurity levels [7], and in the present study PC3 had positive and significant correlation with poverty and GINI, where a higher GINI equals to higher inequality. Poverty and inequality are economic and sociocultural drivers of dietary diversity and quantity and of food systems [54]. Countries where agricultural and transitional patterns appears have less dietary diversity and less kilocalories in their diet when compared to a western diet [10].

The positive and moderate correlation between PC5 and the female labor force can beat least partly explained by a phenomenon called agricultural feminization. The percentage of women working in agriculture is rising in many countries in Africa, Asia, and Latin America, because of greater migration rates of men compared to women. In developing countries, employment opportunities increase in non-farm sectors, and when men move out of agriculture to other activities, women tend to remain on the farm or move out more slowly. Their responsibilities in agriculture may increase in response to this phenomenon. Even in Latin America, where farming has traditionally been a male occupation, the share of women in agricultural employment is increasing when analyzing historical trends [17]. The correlation between PC5 and the female labor force variable in this study was positive in all subgroups of income classification, being higher in countries of middle-income (countries classified as lower and upper middle-income), being only positive and weak in low and high-income countries. The correlation between PC5 and female labor force was 0.10 (95%CI: − 0.32-0.50, *p*-value = 0.65), 0.49 (95%CI: 0.23-0.68, p-value < 0.01), 0.46 (95%CI: 0.20-0.66, p-value < 0.01) and 0.11 (95%CI: − 0.16-0.37, p-value = 0.45) in low-income, lower middle-income, upper middle-income, and high-income country classifications, respectively. Without stratification by income country classification, the correlation between PC5 and female labor force is 0.43, as reported in Table 3.

There is evidence of the nutrition transition occurring in sub-Saharan African countries and India. However, low food diversity can still be a prevailing characteristic of the dietary patterns of these regions [17, 55]. In this sense, the analysis of the median values of each time series may be useful for the purpose of this work, which is to identify dietary patterns in worldwide data and their association with socioeconomic data. However, this approach may not identify some recent trends of food availability.

The agricultural and the coastal dietary patterns identified herein seems to be more associated with cultural and regional differences than with socioeconomic variables. Traditional patterns usually preserve more regional characteristics [8]. Regional patterns in a country level can be labelled with the name of their country, as for example “traditional Japanese” or “traditional Korean” [56].

In the sensitivity analysis, agricultural patterns become more varied as time passed and economic development and growth in income occurred, and this is in concordance with the bennet’s law [45]. In the westernized dietary pattern, sugar and alcoholic beverages begin to appear in the late 80’s and 90’s, characterizing greater processing dietary patterns through time, aligned with previous studies [57]. The coastal dietary pattern remained stable with fish and seafood always present through time.

### Limitations

Food balance sheets do not reflect the actual food consumption of the population. Also, it does not consider losses in food preparation and food preparation methods, leftovers after food consumption, representing only the food that is available for human consumption in each region. Comparing with methods used for assessing food consumption, like the 24-h dietary recall, food balance sheets overestimate the mean regional values for a variety of food groups [3, 4, 58–60].

Low- and middle-income countries at different stages of the nutrition transition are more likely to have a lower quality of food availability data, as they have a marked presence of subsistence agriculture, this one not included in the calculation [61]. In developing countries there may be an underestimation, while in developed countries overestimation of per capita food availability [3, 60]. Also, the method does not capture regional and cultural variations within the country [4].

However, all methods of dietary measurement have limitations [62]. The choice of the appropriate method depends on the purpose of the study. In this sense, per capita food availability method by FAO food balance sheets generates important data for trend assessment over time, being the only one available for many countries in large time ranges and so may be suitable for an ecological study analyzing multiple countries at the same time, given the absence to date of other methods for many countries [4, 63]. Global food consumption databases with data from national country samples are still under development until this date [64].

Another possible limitation of the study is the fact that the analysis was performed in median values of each time series of food availability data. Longitudinal data from 1961 to 2013 (all available years) were used to gain information in this exploratory analysis. They were calculated with the aim to summarize with a robust estimator each time series into one single value for each country and food group, creating a new dataset where statistical analyses (PCA and correlations) were performed to analyze the data. In this sense, each median value of this dataset can be from a different year since it depends on each time series. However, the aim of using medians was only to summarize into one single value the frequency distributions of each time series, to capture general trends in this food availability data available at FAOSTAT, given the exploratory nature of this study. Multivariate statistical analyses in nutrition can be exploratory, and hence dietary patterns identified by these techniques are often called a posteriori dietary patterns [20]. It is of knowledge of the authors that few studies analyzed dietary patterns in an a posteriori manner in food availability data derived from Food Balance Sheets [10, 18, 39].

A posteriori dietary patterns can be labelled subjectively, and the amount of food groups included in the analyses often vary [8]. Even if the analysis is characterized as a posteriori, some prior knowledge is required to label the dietary pattern identified. There is no guarantee that the labels will always have conceptual meanings [52]. However, western is a common label in the literature, being utilized in 44% (*n* = 84) of previously reviewed studies [8]. The label agricultural pattern used here was already used as well in two previous ecological studies using a posteriori statistical analysis to identify dietary patterns in food availability data [10, 39]. But often, labels such as prudent, traditional, healthy, Mediterranean [8], westernized [10], rural, diverse [65], agricultural, transitional [10, 39] or “sweet”, unhealthy, healthy and Mediterranean-like [66] are used, and there is no consensus on how to classify a dietary pattern [8, 66]. Some foods can have a higher factor loading in Factor Analysis (when the multivariate normal distribution assumption is properly respected [24]) in more than one dietary pattern in a study, as for example, in both a western pattern and a traditional pattern [52]. Despite of that, a posteriori dietary patterns are commonly used in the nutritional literature and when analyzing dietary data [8, 20, 52], because individual foods are often correlated with one another [67]. The rationale of this approach is due to the fact that different individual foods are consumed together in complex combinations and not in isolation of each other [8], being often a more realistic analysis of dietary data when compared to a reductionist approach when one nutrient/food/food group is associated with another variable under study [68].

More formally, a western dietary pattern is often composed of foods such as red meat, processed meat, butter, margarine, sugar, refined grains, high-fat dairy, sugary drinks and condiments [20, 66] and some ultra-processed food [69]. Even if the present analysis identify a westernized a posteriori dietary pattern globally, a western diet may not be uniform across regions, but it is known that an increase in the availability of animal origin and energy-rich foods, as well as the level of food processing occurred more generally [4, 5, 7]. An agricultural dietary pattern was already characterized in previous ecological studies by cereals, starchy staples and pulses, and these foods shared more energy in percentage of the whole when compared to other patterns, such as a westernized and a transitional one [10]. In this sense, a transitional dietary pattern can be characterized as one between an agricultural and an western one, having less share of starchy staples and more by foods that composes a western diet, starting to have more food variety [10]. One previous studies used the label “rural” to describe a pattern of rural regions [65].

Given the ecological nature of this study as previously mentioned [10, 39], this can somewhat similar to the ecological fallacy, a well-known epidemiological phenomenon, whereas the observations realized in more general aggregates may not hold to individuals. A potential strategy for reducing ecologic bias is to use smaller units in an ecologic study (e.g., countries instead of states or countries) to make the groups more homogeneous [70]. However, a global food consumption database is still not available until this date for all these countries [64]. Despite of that, foods that characterize a western diet have been identified in western dietary patterns in different contexts, even with heterogeneity of some foods in its composition between studies [10, 20, 39, 52, 66].

Different analytical steps are required to perform multivariate analysis [8, 52]. Reproducibility of a posteriori dietary patterns identified by multivariate statistical techniques have been putted into question. Some patterns can be more heterogeneous than others when compared [8, 53]. A western dietary pattern tends to be similar in different studies, although some variations occur [66].

When performing the statistical analyses performed herein in median values, the effect of time cannot be analyzed properly, and hence the generalization to the nutrition transition is limited. However, the a posteriori dietary patterns identified in this study can be partly explained by the nutrition transition [5] and the westernization of dietary patterns [4, 16] that occurred in the time frame that the data is available. In this sense, the a posteriori dietary patterns identified herein can be explained by the literature.

However, in previous studies, dietary patterns were identified in a transversal manner (one different year per country) and the nutrition transition was cited as a possible explanation for them [10, 39]. Additionally, some correlations between dietary patterns and socioeconomic variables performed in subgroups of country income level may lack statistical power due to low sample sizes. Low-income countries had the smallest sample size of all strata of income levels (*n* = 23). However, these subgroup analyses were performed only with the aim of assessing confounding of income in the association between the identified dietary patterns with the socioeconomic variables.

It is of noteworthy importance that the associations outlined above between the principal components and the socioeconomic variables are also dependent on the unit of the latter (i.e., percentages and not total values for trade liberalization and urban population). Both are also presented in total values in the World Bank [71, 71].

## Conclusion

Dietary patterns were identified in food availability per capita data at the global level after Principal Component Analysis was applied. Five dietary patterns were identified, a westernized dietary pattern, a transitional agricultural dietary pattern, two agricultural dietary patterns and a coastal dietary pattern. The westernized dietary pattern was associated with socioeconomic data that are proxy of economic development, more specifically, income, urbanization, and trade liberalization. This association did not occur for the other dietary patterns identified, these being less driven by economic development and more by regional characteristics, associated with GINI index, poverty, and female labor force. Principal Component Analysis was adequate to identify dietary patterns in food availability data.

## Data Availability

The datasets, statistical analysis and statistical programming supporting the conclusions of this article are available in the Open Science Framework repository, https://osf.io/br4vu/.
